# Determinants of household food security and dietary diversity during the COVID-19 pandemic in Bangladesh

**DOI:** 10.1017/S1368980020005042

**Published:** 2021-04

**Authors:** Satyajit Kundu, Md. Hasan Al Banna, Abu Sayeed, Mst. Sadia Sultana, Keith Brazendale, Jody Harris, Moumita Mandal, Ishrat Jahan, Mohammad Tazrian Abid, Md Shafiqul Islam Khan

**Affiliations:** 1 Department of Biochemistry and Food Analysis, Patuakhali Science and Technology University, Patuakhali, Bangladesh; 2 Department of Food Microbiology, Patuakhali Science and Technology University, Patuakhali, Bangladesh; 3 Department of Post-Harvest Technology and Marketing, Patuakhali Science and Technology University, Patuakhali 8602, Bangladesh; 4 Department of Public Health and Informatics, Jahangirnagar University, Savar, Dhaka, Bangladesh; 5 Department of Health Sciences, University of Central Florida, Orlando, USA; 6 World Vegetable Center, Bangkok, Thailand; 7 Faculty of Nutrition and Food Science, Patuakhali Science and Technology University, Patuakhali, Bangladesh

**Keywords:** Household food security, Household dietary diversity, COVID-19, Bangladesh

## Abstract

**Objectives:**

The study aimed to determine the associated factors of household food security (HFS) and household dietary diversity (HDD) during the COVID−19 pandemic in Bangladesh.

**Design:**

Both online survey and face-to-face interviews were employed in this cross-sectional study. The Household Food Security Scale and Household Dietary Diversity Score were used to access HFS and HDD, respectively. The HDD scores were derived from a 24-h recall of food intake from 12 groups.

**Setting:**

Bangladesh.

**Participants:**

A total sample of 1876 households were recruited.

**Results:**

The overall mean scores of HFS and HDD were 31·86 (sd 2·52) and 6·22 (sd 5·49), respectively. Being a rural resident, having no formal education, occupation of household head other than government job and low monthly income were potential determinants of lower HFS and HDD. Approximately 45 % and 61 % of Bangladeshi households did not get the same quantity and same type of food, respectively, as they got before the pandemic. Over 10 % of respondents reported that they lost their job or had to close their businesses, and income reduction was reported by over 70 % of household income earners during the COVID-19 pandemic, which in turn was negatively associated with HFS and HDD.

**Conclusion:**

Household socio-economic variables and COVID-19 effects on occupation and income are potential predictors of lower HFS and HDD scores. HFS and HDD deserve more attention during this pandemic particularly with reference to low-earning households and the households whose earning persons’ occupation has been negatively impacted during the COVID-19 pandemic.

The COVID-19 pandemic has impacted the health and well-being of the global population and poses a significant threat to the food security and nutrition of millions of people across the world^(1)^. Different preventive measures taken by governments to contain COVID-19 (e.g. staying at home orders, closure of industries, border restrictions, etc.) may pose additional barriers to food systems, including marketing, logistics and trading, which could potentially impact food security^(1)^. Moreover, the economic turmoil as a result of the pandemic has jeopardised physical and economic access to sufficient, and sufficiently nutritious, food^(2)^. Recent evidence shows that prior to the end of the year 2020, food insecurity could nearly double to 265 million people worldwide due to the COVID-19 pandemic^(3)^. Consequently, malnutrition, hunger, low dietary diversity and other nutrition-related problems are likely to increase due to COVID-19^(2)^.

Household food security (HFS) is defined as when all members of a given household have physical and economic access to sufficient, safe and nutritious food at all times that can meet their dietary needs and food preferences for an active and healthy life^(3)^. HFS is underpinned by food availability, accessibility, utilisation and stability^(4)^. The novel COVID-19 pandemic has the ability to impact HFS by not only disrupting food systems but also jeopardising household incomes due to unemployment and physical access to food due to home quarantine orders^(5)^. Several additional factors have been associated with HFS such as socio-economic status, family income, education level and family size^(6,7)^. To tackle the potential impact of COVID-19, information is needed on how factors that influence HFS are currently being affected during the COVID-19 pandemic.

Another key indicator of a household’s ability to not only provide enough food, but a diverse range of foods for good nutrition, is dietary diversity. HDD is a proxy indicator measuring the economic ability of a household to access a variety of foods during a determined period and is widely used as an indicator of food security as a whole^(8)^. Previous studies revealed that higher HDD is associated with socio-economic status and HFS^(8)^. Further determinants, such as type of residency (urban *v*. rural area), type of family (joint family *v*. nuclear family) and occupation have a significant association with adequate HDD^(9)^.

In light of the COVID-19 pandemic, a new set of challenges for households to maintain a healthy and varied diet have arisen^(10)^. Only a handful of studies have explored food security, food system disruption and dietary diversity in the USA (11), China (12), India (29) and Bangladesh (13), during the COVID-19 pandemic. A recent study by Adams *et al.* noted that the number of families from the USA reporting ‘very low’ scores for food security increased by 20 % from data prior to COVID-19^(11)^. Another recent study on dietary diversity among Chinese residents concluded that people living in areas with a high number of confirmed COVID-19 cases had a lower score of HDD^(12)^. Lastly, one Bangladeshi study reported that families’ food insecurity increased by 51·7 % during the COVID-19 home quarantine (lockdown) period^(13)^.

Although Bangladesh has achieved substantial macro-economic growth in the past decades, the COVID-19 pandemic has made a significant impact on households nationwide. A recent nationwide survey by BRAC, a development organisation in Bangladesh, showed that 93 % of participants have suffered a loss of earnings, with 54 % reporting no income in March 2020^(14)^. Moreover, disruption in transportation systems has caused the dumping of perishable food products and dramatic price reductions, affecting food security for rural producers^(15)^. Thus, despite several steps taken by the Bangladeshi government to address food insecurity during the COVID-19, such as relief, distributing cash aid, etc^(16)^, increased poverty and economic turmoil of this magnitude will likely drive many people to short-term food insecurity and poor dietary diversity.

Collectively, there is a lack of information about HFS and HDD amid the COVID-19 pandemic, and an exploration of these issues is urgently needed in Bangladesh. A better understanding of key determinants during the COVID-19 pandemic will help government agencies in the development and implementation of policies and practices that can positively impact food security and improve dietary diversity, in this and future shocks. Thus, this study aimed to explore HFS and household dietary diversity (HDD) status, and its associated factors among Bangladeshi households during the COVID-19 pandemic.

## Methods

### Study setting, design and participants

A community-based cross-sectional survey was conducted to assess the HFS and HDD of the Bangladeshi population from 7 September to 15 September 2020, approximately 5 months after the start of lockdown (e.g. staying at home, closure of industries, border restriction, etc.)^(13)^. The Government of Bangladesh decided in early June to enforce zone-wise lockdowns across the region, dividing areas into three zones (red, yellow and green) based on the number of COVID-19 positive patients. Red, yellow and green were marked as high-, moderate- and low-risk zones, respectively^(17)^. Considering these restrictions, data were collected using online platforms and face-to-face interviews. Online data were collected through social media (e.g. Facebook and WhatsApp) across the country using the snowball sampling method. Face-to-face data were collected from green and yellow zones only. At least one rural and one urban area (green or yellow zones only) from all nine divisions/states were chosen by the authors for face-to-face data collection, based on convenience of access. In total, 18 research staff were involved in face-to-face interviews. Prior to data collection, research staff was trained by the field research supervisor. Considering the limited number of studies on the topic, a 50 % response rate, 95 % confidence level and 2·5 % margin of error were used to calculate the needed sample size of 1537 to achieve 80 % power. In total, 1876 participants replied during the survey duration, and all participants were included in the final analyses. Data from 1164 (62 %) participants were collected randomly via face-to-face interviews, and data from 712 (38 %) participants were collected using online platforms. The eligibility criteria for the study was being a Bangladeshi resident, being an adult (≥ 18 years) and residing in Bangladesh during COVID-19.

### Procedures

The pre-study questionnaire was written in English, and two bilingual researchers translated it into the local language (Bengali). Another independent bilingual expert back-translated the questionnaire to check for consistencies and to prevent any bias. The electronic forms were shared in the local language (Bengali). The survey questionnaire was pilot-tested with a small group (100 households) in both online and face-to-face surveys to ensure its clarity and to avoid any unnecessary/repeated questions. Face-to-face data were collected from the head of the household, whereas online data were collected from any adult member of the household who used internet. The authors distributed the survey link in all nine divisions of Bangladesh. By clicking on the link, the participant was automatically directed to study information and an informed consent page. If they agreed to participate in the survey, the participant had to fill in demographic information, after which, a set of questions followed. To capture households in divisions/states designated as red zone areas, social media avenues were targeted. Face-to-face data collection from door-to-door using appropriate personal protective equipment and maintaining social distance practices was employed in only yellow and green zones. A total of nine rural and nine urban areas were selected based on transport facilities and distances from respective divisional headquarters. All households were selected randomly from the selected areas. The study questionnaire included a short overview of the study context, purpose, procedures, confidentiality agreement and informed consent. The privacy of the electronic data was ensured using an anonymous form and storing data in a password-protected folder.

### Survey contents

The survey consisted of 38 close-ended items, which took respondents approximately 20–25 min to complete. The questionnaire was split into four sections: participant characteristics (10 items), effects of COVID-19 on participants’ day-to-day life (5 items), HFS (11 items) and HDD (12 items). Socio-demographic data were collected on age, gender, educational status, occupation of household head, monthly family income, location of residence, and family size and type. Participants were asked about the effects of COVID-19 on income, occupation, food quality and food quantity. The questions to assess the COVID-19 effects on these variables were closed-ended and coded with numerical scores.

### Measures

#### Household Food Security Scale

We evaluated HFS using the Household Food Security Scale (HHFS), a measure that reflects a household’s food security for the previous month^(18)^. The HHFS consists of 11 items covering the topics of purchased rice and perishable food, cooking frequency, consumption of snacks and management strategies, with a score assigned for each item response^(18)^. Higher and lower scores were assigned for responses which indicated a higher (i.e. more favourable) *v*. lower (i.e. less favourable) food security status, respectively. For example, for the frequency of daily cooking, a score of 1 was assigned to those who responded they typically never cook on a daily basis, which indicated that the household had little-to-no store of food to cook with. For those respondents who reported cooking four or more times per day, a score of 5 was assigned, which indicated that the household had an adequate supply of food to use for cooking^(18)^. A higher score indicated a more favourable HHFS. The reliability of the HHFS was high (Cronbach’s *α* = 0·81).

#### Household Dietary Diversity Score

Household Dietary Diversity Score (HDDS) was calculated by summing up the number of food or food groups eaten over the past 24 h by any member of the household^(19)^. In total, the 12 food groups (FG) were as follows: (FG1) cereals; (FG2) tubers and roots; (FG3) vegetables; (FG4) fruits; (FG5) meat, poultry, organ, etc.; (FG6) eggs; (FG7) fish and others seafood; (FG8) pulses, legumes and nuts; (FG9) milk and other dairy products; (FG10) oils and fats and butter; (FG11) sugar and honey; and (FG12) miscellaneous foods such as condiments and processed foods like snacks, and beverages. The authors’ assigned values for each group as ‘0’ for the negative answer (not consumed), or ‘1’ for the positive answer (consumed). Higher dietary diversity was indicated by a higher score, ranging from 0 to 12. We used the HDDS scale both as a continuous and categorical variable for analyses. For categorical HDD, scores were divided into three categories: high dietary diversity (7–12), medium dietary diversity (4–6) and low dietary diversity (0–3)^(6)^. The internal consistency of the HDDS scales was adequate (Cronbach’s *α* = 0·77).

### Statistical analyses

Descriptive statistics were calculated for socio-demographic characteristics of the participants and variables assessing the impact of COVID-19 on respondents’ day-to-day life. Bivariate linear regression was employed to assess the associations between socio-demographic characteristics, the variables related to the impact of COVID-19 effect on daily life, HDD and HFS. Multicollinearity was checked via the variance inflation factor. The final model selection was computed using Hosmer and Lemeshow goodness-of-fit test, and the significance of variables was assessed using the Wald test. The estimates of the strengths of associations were demonstrated by the *β* (co-efficient) with a 95 % CI. All tests were two-tailed, and a *P*-value of <0·05 was set to determine statistical significance. Statistical analyses were performed using SPSS version 23.0 (IBM SPSS Statistics).

## Results

The majority of the family/household heads were male (93·6 %), and above 40 years old (64·7 %). Nearly, half (45·9 %) of household heads were educated below the secondary level and 20·3 % were self-employed. The majority of the households were from rural areas (59·1 %), nuclear family (81·1 %), with a family size of ≤ 5 (65·5 %) and a monthly income of ≤ 20 000 BDT (238$) (65·4 %). Overall, 41·2 % of households had no refrigerator and 36·1 % of household heads reported getting their dietary/nutrition information from traditional media sources, such as television and radio.

### Household dietary diversity score and household food security scores of participants

In the present study, the mean HDD score during the COVID-19 pandemic was 6·22 (sd 2·52). When HDD scores were split into the three categories (high/medium/low dietary diversity), 779 (41·5 %) households reported high dietary diversity, 842 (44·9 %) households had medium dietary diversity and 255 (13·6 %) households had low dietary diversity. The mean HFS score during the COVID-19 pandemic was 31·86 (sd 5·49). Table 1 represents the proportions of participants who consumed different food groups in the previous 24 h. The cereals group (100 %) had the highest consumption rate, followed by the vegetable group (79·9 %), with pulses, legumes and nuts (25·4 %), and oils and fats (26·9 %), the least reported consumption groups (Table 1).

**Table 1:** Food groups consumption by Bangladeshi households during COVID-19 pandemic in the last 24 h (*n* 1876)

	Consumed		Not consumed	
Food groups	*n*	%	*n*	%
Cereals (FG1)	1876	100	0	0·0
Tubers and roots (FG2)	1289	68·7	587	31·3
Vegetables (FG3)	1499	79·9	377	20·1
Fruits (FG4)	962	51·3	914	48·7
Meat, poultry, organ, etc. (FG5)	701	37·4	1175	62·6
Eggs (FG6)	966	51·5	910	48·5
Fish and other sea food (FG7)	846	45·1	1030	54·9
Pulses, legumes and nuts (FG8)	476	25·4	1400	74·6
Milk and other dairy products (FG9)	716	38·2	1160	61·8
Oils, fats and butter (FG10)	505	26·9	1371	73·1
Sugar and honey (FG11)	651	34·7	1225	65·3
Miscellaneous*(FG12)	1182	63·0	694	37·0

* Miscellaneous foods include condiments and most processed foods like snacks, beverages, tea, coffee, etc.

### Association of socio-demographic variables with household dietary diversity and household food security score

Households from rural areas had lower HHD scores (*β* = −2·08, 95 % CI: −2·29, −1·87) and HFS scores (*β* = −4·68, 95 % CI: −5·14, −4·22) compared to households from urban areas. Household heads’ higher educational levels (below secondary, above secondary and a university graduate or above) were associated with increased scores on both the HDD and HFS, compared to the head of a household having no formal schooling. Households with a family head or main household earner who was involved in a private job, business, day labouring, farming or other occupation were significantly associated with lower HDD scores and HFS scores, compared to the families having a family head or main household earner who was involved in a government job. Households with a monthly income of ≤ 20 000 BDT had lower HDD scores (*β* = −3·04, 95 % CI: −3·24, −2·85) and HFS scores (*β* = −5·61, 95 % CI: −6·07, −5·16) compared to households with a monthly income of above 20 000 BDT. Family members getting dietary/nutrition information from online resources had significantly higher HDD scores (*β* = 1·57, 95 % CI: 1·07, 2·07), and families who did not know any dietary/nutrition information had lower HDD scores (*β* = −1·63, 95 % CI: −2·11, −1·16), compared to families who got dietary/nutrition information from health professionals (Table 2).

**Table 2:** Associations between socio-demographic variables, household dietary diversity scores and household food security scores of Bangladesh households (*n* 1876)

Variables	*n*	%	Dietary diversity				Food security			
			*R* ^2^	*AR* ^2^	*β*	95 % CI	*R* ^2^	*AR* ^2^	*β*	95 % CI
Residence										
Rural	1108	59·1	0·165	0·165	−2·08***	−2·29, −1·87	0·176	0·175	−4·68***	−5·14, −4·22
Urban	768	40·9			Ref.	Ref.			Ref.	Ref.
Age of family head										
≤40 years	662	35·3	0·016	0·015	Ref.	Ref.	0·020	0·019	Ref.	Ref.
>40 years	1214	64·7			0·66***	0·42, 0·90			1·61***	1·09, 2·12
Gender of family head										
Male	1765	93·6	0·000	0·000	0·23	−0·24, 0·69	0·001	0·001	0·80	−0·22, 1·81
Female	120	6·4			Ref.	Ref.			Ref.	Ref.
Education of family head										
No schooling	208	11·1	0·338	0·337	Ref.	Ref.	0·262	0·261	Ref.	Ref.
Below secondary	862	45·9			0·64***	0·33, 0·95			2·11***	1·40, 2·83
Above secondary	225	12·0			2·34***	1·95, 2·72			6·23***	5·34, 7·12
Graduate or above	581	31·0			3·72***	3·39, 4·04			7·54***	6·80, 8·29
Occupation of family head										
Govt. job	307	16·4	0·379	0·377	Ref.	Ref.	0·327	0·325	Ref.	Ref.
Private job	253	13·5			−1·42***	−1·75, −1·08			−2·68***	−3·43, −1·93
Business	380	20·3			−1·84***	−2·14, −1·54			−2·80***	−3·48, −2·12
Day labourer	343	18·3			−4·55***	−4·86, −4·25			−9·48***	−10·17, −8·78
Farmer	265	14·1			−4·04***	−4·36, −3·71			−6·56***	−7·30, −5·81
Other*	234	12·5			−2·29***	−2·62, −1·95			−3·88***	−4·65, −3·11
Jobless/retired	94	5·0			−3·22***	−3·68, −2·76			−7·10***	−8·15, −6·06
Family type										
Joint	355	18·9	0·004	0·004	Ref.	Ref.	0·009	0·008	Ref.	Ref.
Nuclear	1521	81·1			0·41**	0·12, 0·70			1·31***	0·68, 1·95
Family member										
≤ 5 person	1229	65·5	0·005	0·004	Ref.	Ref.	0·008	0·008	Ref.	Ref.
> 5 person	647	34·5			−0·37**	−0·61, −0·13			−1·05***	−1·57, −0·53
Family income (monthly)										
≤ 20 000 BDT	1227	65·4	0·330	0·330	−3·04***	−3·24, −2·85	0·236	0·236	−5·61***	−6·07, −5·16
> 20 000 BDT	649	34·6			Ref.	Ref.			Ref.	Ref.
Having refrigerator										
Yes	1103	58·8	0·348	0·348	3·02***	2·83, 3·20	0·353	0·353	6·63***	6·22, 7·04
No	773	41·2			Ref.	Ref.			Ref.	Ref.
Sources of dietary/nutrition information										
Health professional†	114	6·1	0·167	0·165	Ref.	Ref.	–	–	–	–
Traditional media‡	677	36·1			0·51	−0·05, 0·96			–	–
Online resources	281	15·0			1·57***	1·07, 2·07			–	–
Others§	381	20·3			0·06	−0·42, 0·54			–	–
Don’t know	423	22·5			−1·63***	−2·11, −1·16			–	–

*R*^2^ = *R*-squared, *AR*^2^ = adjusted *R*-squared.

* Others included outsourcing, rickshaw pulling, etc.

† Health professional included physician, dietitian, nutritionist, etc.

‡ Traditional media included TV, radio, newspaper, etc.

§ Others included friends, family members, books, etc.

** *P* < 0·01.

*** *P* < 0·001.

### Variables related to COVID-19 impact on daily life, and household dietary diversity and household food security

A household head or main earner for the household who lost their main occupation due to COVID-19 had a significant negative association with HDD scores (*β* = −1·78, 95 % CI: −2·16, −1·39) and HFS scores (*β* = −4·61, 95 % CI: −5·43, −3·79). Similar results were seen for those individuals who had to change their occupation; lower HDD (*β* = −0·87, 95 % CI: −1·24, −0·50) and HFS (*β* = −2·83, 95 % CI: −3·62, −2·04) scores were observed compared to those who had retained their previous occupation during the COVID-19 pandemic. The households whose monthly income decreased as a result of the COVID-19 pandemic had lower HDD scores (*β* **= −**2·50, 95 % CI: −2·74, −2·27) and HFS scores (*β* = −4·54, 95 % CI: −5·07, −4·01) compared to those with same income before the pandemic. Households who reported that they faced increased food prices due to COVID-19 had significant negative associations with HDD (*β* = −0·95, 95 % CI: −1·43, −0·48) and HFS (*β* = −1·77, 95 % CI: −2·82, −0·73) scores. Families that did not get the same amount of food as before COVID-19 had a significant negative association with HDD score (*β* = −2·25, 95 % CI: −2·45, −2·04) and HFS score (*β* = −5·41, 95 % CI: −5·85, −4·98) compared to those that got the same amount of food as before COVID-19. Similarly, families that did not get the same type of food as they did prior to COVID-19 had a significant negative association with HDD (*β* = −2·74, 95 % CI: −2·94, −2·54) and HFS (*β* = −5·52, 95 % CI: −5·97, −5·08) scores compared to those households/families that were able to purchase the same types of food as they did prior to COVID-19 (Table 3).

**Table 3:** Association between the impact of COVID-19 pandemic, the household dietary diversity scores and household food security scores of Bangladesh households (*n* 1876)

Variables	*n*	%	Dietary diversity				Food security			
			*R* ^2^	*AR* ^2^	*β*	95 % CI	*R* ^2^	*AR* ^2^	*β*	95 % CI
Effect of COVID-19 on occupation of HH/earning person of family										
Sane as before	1507	80·3	0·049	0·048	Ref.	Ref.	0·076	0·075	Ref.	Ref.
Lost job/business closed	192	10·2			−1·78***	−2·16, −1·39			−4·61***	−5·43, −3·79
Occupation switched	177	9·4			−0·87***	−1·24, −0·50			−2·83***	−3·62, −2·04
Effect of COVID-19 on income of HH/earning person of family										
Sane as before	489	26·1	0·195	0·194	Ref.	Ref.	0·137	0·136	Ref.	Ref.
Less than before	1347	71·8			−2·50***	−2·74, −2·27			−4·54***	−5·07, −4·01
More than before	40	2·1			0·50	−1·23, 0·23			−0·27	−1·92, 1·37
Increase of food prices due to COVID-19										
Yes	1763	94·0	0·008	0·008	−0·95***	−1·43, −0·48	0·006	0·005	−1·77**	−2·82, −0·73
No	0	0·0			–	–			–	–
Don’t know	113	6·0			Ref.	Ref.			Ref.	Ref.
Get same amount of food as before COVID-19										
Yes	1026	54·7	0·197	0·197	Ref.	Ref.	0·241	0·240	Ref.	Ref.
No	850	45·3			−2·25***	−2·45, −2·04			−5·41***	−5·85, −4·98
Get same type of food as before COVID-19										
Yes	732	39·0	0·281	0·281	Ref.	Ref.	0·241	0·240	Ref.	Ref.
No	1144	61·0			−2·74***	−2·94, −2·54			−5·52***	−5·97, −5·08

*R*^2^ = *R*-squared; *AR*^2^ = adjusted *R*-squared; HH = household head.

** *P* < 0·01.

*** *P* < 0·001.

## Discussion

This study explored the determinants of HFS and HDD in Bangladesh during the COVID-19 pandemic. This is one of the first studies that has provided the data on HFS and HDD status during the COVID-19 pandemic. We found that both household socio-demographic variables and COVID-19-mediated factors were associated with HFS and HDD. The current COVID-19 pandemic has negatively impacted the food security and diet quality of millions of people around the globe^(5,20)^. The present study provides evidence of COVID-19’s impact on HDD and food security in a low-resource setting such as Bangladesh. Specifically, results from the current study show that the COVID-19 has impacted the economic status of many families, which in turn negatively affects their food security and dietary diversity. A recent Bangladeshi study provided evidence on the reduction of food insecurity during the COVID-19 lockdown compared to the pre-lockdown period^(13)^.

### Demographic determinants of household food security

One of the main findings from our study was the association between rural residence and lower food security scores. A recent report revealed a 15 % decrease in rural populations consuming three meals per day during COVID-19 compared to the pre-COVID-19 period^(21)^. Comparatively, respondents from lower socio-economic status groups (e.g. day labourers, farmers) and households that do not have a refrigerator had lower food security scores compared to their counterparts in the present study. Occupations other than government jobs do not have or have fewer unemployment benefit or wage compensation services and are unable to do their job from home, so their occupation can be affected by the lockdown, that may result in food insecurity^(5)^. This finding was similar to prior studies of Bangladeshi residents before the pandemic that has shown households with lower socio-economic status have fewer capabilities and resources to produce their food, which can lead to an increase in food insecurity^(7)^. A report during COVID-19 showed a 27 % decrease in per capita food expenditure in lower poverty regions^(21)^.

Higher educational status was positively associated with food security, which might be attributable to the fact that better-educated people can diversify their work environments and, through higher earning potential, have the ability to maintain their families’ food availability^(6)^. However, families with older family heads had more financial security, which might be explained by the fact that older people are more experienced in terms of working, and thus, have the means to ensure their families having stable access to a variety of food. Moreover, nuclear families had significantly higher food security. This finding suggests that higher household size contributes to lower HFS, which is similar to a previous Bangladeshi study^(6)^.

### Demographic determinants of household dietary diversity

The mean HDD score of participants was 6·22 (sd 2·52), but was lower for households in rural areas and with less secure incomes. which is noticeably lower than a recent study in China during COVID-19 (9·7 (sd 2·1))^(12)^, but somewhat similar to a study during the pre-COVID-19 period in India (6·28 (sd 1·3))^(9)^. Our data show that economic stresses imposed by COVID-19 play a significant role in reducing dietary diversity for the households reporting that the income of their household head was affected by COVID-19. This persistent low dietary diversity of many Bangladeshi households during COVID-19 implies the importance of tracking diet-related policies and practices (e.g. access to a variety of foods) during times where households are under additional strain, such as the COVID-19 pandemic. Our study revealed lower dietary diversity among households whose family heads were day labourers, private jobholders, businessman, farmers and others indicating a deficiency in dietary diversity among precariously employed households^(22)^. Individuals who are self-employed, such as day labourers, do not have access to unemployment benefit or wage compensation systems, and their work is not conducted from home; therefore, due to restrictions on residents travel during COVID-19, their employment situation can be impacted resulting in less income to purchase foods^(5)^. Respondents having less family income and whose family income was affected by COVID-19 experienced lower dietary diversity. This finding is supported by previous evidence that spending on food consumption is positively linked to greater diversity^(22)^.

Furthermore, rural residence was associated with lower HDD scores in our study population, corresponding to other previous studies^(12,23)^. It is plausible that lower nutritional knowledge and awareness might lead rural people to less dietary diversity than urban people^(24)^. Furthermore, online ordering and delivery services, as well as consumption of junk foods, are common practices in urban areas which can increase dietary diversity albeit with nutritionally negative foods, among urban people: It has been shown before that dietary diversity increases with increased intake of junk food^(25)^.

Higher educational status of the household head was significantly associated with higher dietary diversity scores, which may be due to improved nutritional awareness in the household^(26)^. Farmer as family heads’ occupation had a significant association with low HDD scores. It is worth to mention that over 80 % of Bangladesh’s total population lives in rural areas and relies directly or indirectly on agriculture^(27)^. In rural areas, there is potential for farming households to lose access to food from their production if they fall sick from COVID-19, are unable to access manpower and materials (e.g. fertilisers and seeds), or lose access to markets to sell their produce due to restrictions on trade and mobility^(5)^. In our study, we found that the nuclear family had more dietary diversity compared to the joint family which is opposite to a previous finding^(9)^ and so likely depends on context.

### Impacts of COVID-19 on household food security

In the present study, 71·8 % of respondents claimed that their income decreased due to COVID-19, and this was positively associated with household food insecurity. This finding aligns with another study indicating that approximately half of a families’ income fell below $1·90 (an indicator of extreme poverty) per day during COVID-19, which was associated with food insecurity^(13)^. Another recent study revealed that 65·35 % and 74·68 % of wage earners had no earning source and did not receive a salary during COVID-19^(28)^. A study in India during the COVID-19 reported that 60 % of surveyed farms’ income dropped by half^(29)^. This precarious situation might play a significant role behind food insecurity in a low-resource, high-agriculture country like Bangladesh.

Moreover, unemployment due to COVID-19 was significantly associated with lower food security scores, again suggesting that reduced income and poverty increased food insecurity during COVID-19^(13)^. Collectively, these findings underscore the impact of economic stress on food access during shocks such as the pandemic^(30)^. The World Food Program predicts that the worldwide pandemic has the capability of increasing food insecurity to 265 million people^(3)^, thus, careful consideration of this situation is required as household food insecurity can also lead to negative health outcomes, such as maternal anxiety and depression^(31,32)^.

### Impacts of COVID-19 on household dietary diversity

Notably, 45·3 % and 61 % of the respondents reported that they did not get the same quantity or type of food as they did prior to COVID-19, respectively. These findings are similar to a study in India where 62 % of respondents reported that their diet was disrupted due to COVID-19^(29)^. Disrupted production and distribution of food due to COVID-19 have clear consequences such as decreased food diversity^(29,33)^. In addition, increased food prices (94 %) due to COVID-19 decrease dietary diversity to a large extent, which has been found in previous studies pre-COVID-19^(5)^.

### Strengths and limitations

This study had several strengths. To the best of our knowledge, it was the first study among Bangladeshi residents that measured HFS and HDD during the COVID-19 pandemic. In addition, this study employed both face-to-face and online surveys for recruitment and sampling to ensure a broad reach of survey responses given the restrictions placed on the country due to the pandemic. Lastly, piloting the surveys prior to data collection added appropriateness and clarity to the survey tools for the context of the study and setting.

However, this study has some limitations. This study mainly includes the online users which may suggest sampling bias by unintentionally excluding those who do not have internet access. This may reduce the generalisability of the findings. In addition, due to the cross-sectional nature of the study, we were unable to evaluate any seasonal variation of HFS and HDD during the COVID-19 pandemic. Although the HDD scores can help determine food accessibility, they do not capture the amount of actual food consumption by households, and nor do they capture changes or reductions in diversity within food groups, which is important for nutrition. Lastly, all information was self-reported by the participants and may be open to reporting bias.

## Conclusion

This study explored the socio-demographic and pandemic-mediated determinants of HFS and dietary diversity during the COVID-19 pandemic in Bangladesh. Rural residence, no formal education, occupation other than government job and low monthly income, loss of occupation and income due to the COVID-19 pandemic, and increased food prices are the major risk factors for low HFS and HDD scores. There is a need for more attention towards HFS and HDD during the COVID-19 pandemic, to maintain food security and health. Authorities should work to protect supply chains of food items in all parts of the community. Food and nutrition assistance needs to be a fundamental part of social protection programmes to ensure diverse food access for the most vulnerable populations by protecting their purchasing power and by directly providing food where necessary. COVID-19 awareness programmes should include HFS and dietary diversity during the pandemic.

## References

[1] UN (2020) Policy Brief: The Impact of COVID-19 on Food Security and Nutrition. https://www.un.org/sites/un2.un.or/file/sg_policy_brief_on_covid_impact_on_food_security.pdf (accessed June 2020).

[2] FAO (2020) Impacts of coronavirus on food security and nutrition in Asia and the Pacific: building more resilient food systems. http://www.fao.org/3/ca9473en/CA9473EN.pdf (accessed September 2020).

[3] WFP (2020) COVID-19 will double number of people facing food crises unless swift action is taken. https://www.wfp.org/news/covid-19-will-double-number-people-facing-foodcrises-unless-swift-action-taken (accessed September 2020).

[4] FAO (2008) An introduction to the basic concepts of food security. FAO Food Security Programme. Rome: Author.

[5] Devereux S , Béné C , Hoddinott J (2020) Conceptualising COVID-19’s impacts on household food security. Food Secur 12, 769–772. 10.1007/s12571-020-01085-0 PMC7358330 32837651

[6] Ahmed JU , Mozahid MN , Dhar AR et al. (2019) Food security and dietary diversity of tea workers of two tea gardens in greater Sylhet district of Bangladesh. GeoJournal, 1–13.

[7] Saha KK , Frongillo EA , Alam DS et al. (2009) Household food security is associated with growth of infants and young children in rural Bangladesh. Public Health Nutr 12, 1556–1562. 19232147 10.1017/S1368980009004765

[8] Huluka AT & Wondimagegnhu BA (2019) Determinants of household dietary diversity in the Yayo biosphere reserve of ethiopia: an empirical analysis using sustainable livelihood framework. Cogent Food Agric 5, 1690829.

[9] Mukherjee A , Paul S , Saha I et al. (2018) Dietary diversity and its determinants: a community-based study among adult population of Durgapur, West Bengal. Med J 11, 296.

[10] Naja F & Hamadeh R (2020) Nutrition amid the COVID-19 pandemic: a multi-level framework for action. Eur J Clin Nutr 74, 1–5. 10.1038/s41430-020-0634-3 PMC7167535 32313188

[11] Adams EL , Caccavale LJ , Smith D et al. (2020) Food insecurity, the home food environment, and parent feeding practices in the era of COVID-19. Obesity 28, 2056–2063. 32762129 10.1002/oby.22996 PMC7436743

[12] Zhao A , Li Z , Ke Y et al. (2020) Dietary diversity among Chinese residents during the COVID-19 outbreak and its associated factors. Nutrients 12, 1699. 10.3390/nu12061699 PMC7352896 32517210

[13] Hamadani JD , Hasan MI , Baldi AJ et al. (2020) Immediate impact of stay-at-home orders to control COVID-19 transmission on socioeconomic conditions, food insecurity, mental health, and intimate partner violence in Bangladeshi women and their families: an interrupted time series. Lancet Glob Health 8, e1380–e1389. 32857955 10.1016/S2214-109X(20)30366-1 PMC7447230

[14] BRAC (2020) Rapid Perception Survey on COVID19 Awareness and Economic Impact (Final Draft). Dhaka, Bangladesh: BRAC.

[15] FAO (2020) *Coronavirus Disease* 2019 (COVID-19). Rome: FAO; available at 10.4060/ca9192en (accessed September 2020).

[16] Chakrobarty S , Rasheduzzaman M , Basunia AK (2020) *COVID-19 and Bangladesh: A Review of Food Security Status and Impact on Society*. 10.2139/ssrn.3665699 (accessed September 2020).

[17] AA (2020) *Bangladesh: Zone-Coded Lockdown as Infections Rise*. Bangladesh: AA; available at https://www.aa.com.tr/en/asia-pacific/bangladesh-zone-coded-lockdown-as-infections-rise/1876571 (accessed September 2020).

[18] Frongillo EA , Chowdhury N , Ekström E-C et al. (2003) Understanding the experience of household food insecurity in rural Bangladesh leads to a measure different from that used in other countries. J Nutr 133, 4158–4162. 14652365 10.1093/jn/133.12.4158

[19] Swindale A & Bilinsky P (2006) Development of a universally applicable household food insecurity measurement tool: process, current status, and outstanding issues. J Nutr 136, 1449S–1452S. 16614442 10.1093/jn/136.5.1449S

[20] United Nations (2020) Policy brief: the impact of COVID-19 on food security and nutrition. https://ec.europa.eu/knowledge4policy/publication/policy-brief-impact-covid-19-food-security-nutrition_en (accessed September 2020).

[21] Rahman HZ & Matin I (2020) Livelihoods, Coping, and Support during the Covid-19 Crisis. Dhaka: BRAC Institute Government Development.

[22] Rabbani A (2014) Household food security in Bangladesh: going beyond poverty measures. Bangladesh Dev Stud 37, 103–125.

[23] Andrissi L , Mottini G , Sebastiani V et al. (2013) Dietary habits and growth: an urban/rural comparison in the Andean region of Apurimac, Peru. Ann Ist Super Sanita 49, 340–346. 24334777 10.4415/ANN_13_04_04

[24] Saeidlou SN , Babaei F & Ayremlou P (2016) Nutritional knowledge, attitude and practice of north west households in Iran: is knowledge likely to become practice? Maedica (Buchar) 11, 286. PMC5543519 28828044

[25] Bezerra IN & Sichieri R (2011) Household food diversity and nutritional status among adults in Brazil. Int J Behav Nutr Phys Act 8, 22. 21439090 10.1186/1479-5868-8-22 PMC3076222

[26] Samuel S , Darebo T , Desta DT et al. (2020) Socio-economic and dietary diversity characteristics are associated with anemia among pregnant women attending antenatal care services in public health centers of Kembata Tembaro Zone, Southern Ethiopia. Food Sci Nutr 8, 1978–1986. 32328264 10.1002/fsn3.1485 PMC7174199

[27] BBS (2019) *Statistical Yearbook of Bangladesh*. Dhaka: Bangladesh Bureau of Statistics. http://bbs.portal.gov.bd/sites/default/files/files/bbs.portal.gov.bd/page/b2db8758_8497_412c_a9ec_6bb299f8b3ab/2020-09-17-15-30-d0e641b2e659019f2aa44cbaf628caa8.pdf (accessed September 2020).

[28] Sultana MS , Khan AH , Hossain S et al. (2020) The association between financial hardship and mental health difficulties among adult wage earners during the COVID-19 pandemic in Bangladesh: findings from a cross-sectional analysis. PsyArXiv. Published online: 12 September 2020. doi: 10.31234/osf.io/q3ehv. PMC8488168 34616314

[29] Harris J , Depenbusch L , Pal AA et al. (2020) Food system disruption: initial livelihood and dietary effects of COVID-19 on vegetable producers in India. Food Secur 12, 841–851. 10.1007/s12571-020-01064-5 PMC7358322 32837650

[30] Global Nutrition Report (2020) The 2020 Global Nutrition Report in the context of Covid-19. 2020. https://globalnutritionreport.org/reports/2020-global-nutrition-report/2020-global-nutrition-reportcontext-covid-19/ (accessed September 2020).

[31] Hadley C & Patil CL (2006) Food insecurity in rural Tanzania is associated with maternal anxiety and depression. Am J Hum Biol Off J Hum Biol Assoc 18, 359–368. 10.1002/ajhb.20505 16634017

[32] Taaffe McLearn K , Minkovitz CS , Strobino DM et al. (2006) Maternal depressive symptoms at 2 to 4 months post partum and early parenting practices. Arch Pediatr Adolesc Med 160, 279–284. 16520447 10.1001/archpedi.160.3.279

[33] World Bank (2020) *World Bank Brief: Food security and Covid-19*. https://www.worldbank.org/en/topic/agriculture/brief/food-security-and-covid19 (accessed September 2020).

